# Preparation and Properties of Rubber Blends for High-Damping-Isolation Bearings

**DOI:** 10.3390/polym11081374

**Published:** 2019-08-20

**Authors:** Tuo Lei, Yong-Wang Zhang, Dong-Liang Kuang, Yong-Rui Yang

**Affiliations:** 1 School of Civil Engineering, Chang′an University, Xi′an 710061, China; 2 School of Civil Engineering, Southeast University, Nanjing 210096, China; 3 School of Materials Science and Engineering, Chang′an University, Xi′an 710064, China

**Keywords:** composite materials, mechanical properties, damping properties, stability

## Abstract

To improve the energy dissipation capacity of rubber isolation bearings, it is important to find a new rubber material with good applicability and high damping properties. Two types of blends were prepared using nitrile rubber (NBR), brominated butyl rubber (BIIR) and ethylene-vinyl acetate copolymer (EVA): NBR/BIIR and NBR/BIIR/EVA. The vulcanization, mechanical and damping properties of the blends were analyzed. The results show that both blends exhibit excellent vulcanization plateaus and mechanical properties. For NBR/BIIR, as the BIIR content increases, the complementary effects of NBR and BIIR afforded by blending are enhanced. Two damping peaks appeared in the tanδ-T curve and shifted toward lower and higher temperatures, respectively, which clearly widened the effective damping temperature range. However, the damping value in the valley of the tanδ-T curve was as low as 0.39. For NBR/BIIR/EVA, the addition of EVA greatly increased damping in the valley of the tanδ-T curve to approximately 0.54. EVA was observed to be the optimal polymer for improving the compatibility of the NBR/BIIR blend. Moreover, hot air thermal aging tests showed that both blends demonstrated good stability.

## 1. Introduction

As a highly unexpected and unpredictable natural disaster, earthquakes do serious damage to human lives and properties. Isolation bearings, an important device to reduce the seismic force transmitted to buildings with functions of energy dissipation, are set between the foundation and the building structure to increases the structure deformability and hysteretic damping ability of buildings, thereby protecting life and reducing economic loss [1,2,3]. In recent years, with the development and application of seismic isolation technology, the demand for high-damping seismic isolation bearings for buildings has become increasingly urgent [4]. Building seismic isolation bearings require not only high vertical bearing capacity but also good energy dissipation capacity and stability when large horizontal shear deformation occurs [5]. Excellent damping performance is a prerequisite for the isolation bearing materials. Compared with normal laminated rubber bearings, high-damping-isolation bearings usually exhibit better damping performance due to the use of composite rubber materials with high damping properties. Meanwhile, the bearings retain the horizontal and vertical mechanical properties of normal rubber bearings [6]. The composite materials used directly affect the mechanical properties, damping performance and durability of the bearings [7,8,9]. An ideal high-damping composite material should have excellent mechanical properties, a high dissipation factor, good anti-aging stability, and a wide effective damping temperature range [10,11,12]. Therefore, finding a suitable high-damping rubber material is the key to preparing high-damping-isolation bearings.

Generally, the blending of two or more types of rubbers is a useful and important way for the preparation and development of rubber blends with properties superior to those of individual constituents, which is also beneficial for improving damping and physical properties [13,14]. In recent years, much attention has been paid to prepare wide temperature range of damping materials by blending. Studies on the damping properties of chlorinated butyl rubber (CIIR) and nitrile rubber (NBR) blends by Tao et al. [15] indicate that the effective damping temperature range is approximately 130 °C. However, the compatibility of the two-phase materials is not particularly high, and the crosslink densities of the two phases are different. The damping value in the valley of the tanδ-T curve is low. The damping properties of natural rubber (NR)/butyl rubber (IIR) blends compatibilized by isobutylene-isoprene block copolymer (IIBC) were researched by Li et al. [16]. The results showed that the NR/IIR blends have typical incompatible sea island biphase structure and damping peak height is decreased while the damping temperature range is broadened. Furthermore, most of the modifications of blends by rubber blending focus on the improvement of damping properties, and only a few studies deal with the mechanical and aging properties of rubber blends, which is not enough research considering materials used for isolation bearings must have a good overall performance.

Among damping materials, polymer composites—especially the interpenetrating polymer networks (IPNs)—are extensively used as damping materials, for their high viscoelastic property around the glass transition temperature [17,18,19,20]. For example, Huang et al. [21] studied the damping properties of polydimethylsiloxane (PDMS) and polymethacrylate (PAC) sequential interpenetrating polymer networks. The results indicated that forced compatibility, resulting from the multilayer network from IPNs and the PDMS/PAC vulcanization system, is the key to obtaining a broad damping functional region. The physical and damping properties of pure polyurethane (PU)/epoxy resin (EP) graft interpenetrating polymer network (IPN) composites modified from introducing multi-walled carbon nanotubes (CNTs), hydroxy-terminated liquid nitrile rubber (HTLN) and nature clay montmorillonite (MMT) were further researched by Chen et al. [22,23,24,25]. Results reveal that the addition of CNTs, HTLN and MMT can improve the damping properties, tensile strength and impact strength of PU/EP IPN. Though there exist attractive properties of the IPN, the effective damping temperature range is still narrower for some extreme environments. In addition, complexity of the synthesis process and high cost of INP composites greatly limit the application of INP damping materials as structural damping materials in engineering applications (i.e., high-damping-isolation bearings).

The other way utilizes the introduction of other energy dissipation methods, such as the breaking and reorganizing of reversible process [26], the introduction of relaxation components [27] and so on. For example, Wu et al. [28] added hindered phenol into chlorinated polyethylene (CPE) and found a novel transition peak induced by the intermolecular hydrogen bonding. Zhao et al. [29] studied the damping and mechanical properties of hindered phenol AO-80/CIIR/NBR blend, and noted that the addition of AO-80 can effectively improve the damping in the valley of the blend materials. Qiao et al. [30] have systematically expounded the interaction mechanism of the significantly improved damping property of NBR contributed by the introduction of small molecules of AO-80, which provided some useful information to design the high-performance damping materials by adding small molecules. After that, abundant research based on hydrogen bonds has been implemented to prepare high damping material [31,32,33]. The introduction of hydrogen bonds can effectively improve the damping peak height, but the effective damping temperature range is slightly broadened. Besides, the damping stability is deteriorated due to the phase separation of matrix and additives.

Among damping rubbers, butyl rubber and NBR are most commonly used. BIIR is a type of modified halogenated butyl rubber that maintains the damping properties of butyl rubber while exhibiting better curing activity and polarity [34,35]. However, the rubber glass transition temperature (*T*_g_) is approximately −40 °C, and the effective damping temperature range (corresponding to the temperature range in which tanδ ≥ 0.3 [36]) is mainly concentrated at low temperatures. NBR is known to exhibit numerous outstanding properties such as desirable physical and mechanical properties, and excellent aging resistance performance. The *T*_g_ of NBR is approximately −10 °C, and its damping performance is also excellent. As a damping material, NBR is suitable for high-temperature environments [37,38,39]. Considering the large *T*_g_ of NBR, the introduction of NBR into BIIR should be an effective way to improve the high-temperature damping property of BIIR (i.e., a wide effective damping temperature range). However, NBR/BIIR blends are slightly soluble and usually exhibit phase-separated morphology and poor interfacial adhesion between the phases. Therefore, it is necessary to introduce an interfacial compatibilizer to the binary system to improve the total performance. Ethylene-vinyl acetate copolymer (EVA) is a copolymer of non-polar ethylene (E) monomer and a polar vinyl acetate (VA) monomer, which contains both a non-polar ethylene segment and a polar vinyl acetate segment in its molecular chain [40,41,42,43]. It can be added as a compatibilizer to the NBR/BIIR blends to reduce the surface tension and improve the compatibility of the two phases. The EVA has the potential to improve the mechanical and damping properties simultaneously [44,45,46].

In the following section, the blending of strongly polar NBR and weakly polar BIIR to prepare NBR/BIIR blends is described in detail first. The vulcanization, mechanical and damping properties of the blends were analyzed using a moving die rheometer, a universal testing machine, and a dynamic mechanical analyzer. Based on the optimal blend ratio of NBR/BIIR, EVA copolymer, a material compatible with NBR/BIIR, was added to prepare NBR/BIIR/EVA. The same properties of NBR/BIIR/EVA were also systematically investigated. Furthermore, the aging properties of the two blends were studied using a hot air thermal aging test chamber. Through a comparison of the test results, the optimal mixture ratio of NBR/BIIR/EVA was determined, and all properties meet the requirements of the standard (Chinese Standard JG/T-118-2019). The results provide a theoretical basis for synthesizing composite rubber materials for high-damping-isolation bearings.

## 2. Experimental Research

### 2.1. Materials

**Figure 1:**
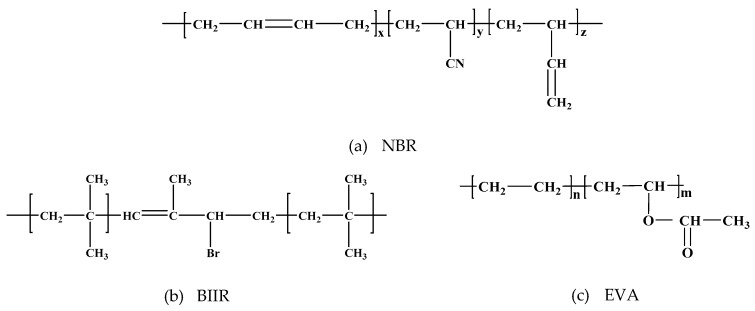
Molecular structural formulas: (**a**) nitrile rubber (NBR); (**b**) brominated butyl rubber (BIIR); (**c**) ethylene-vinyl acetate (EVA).

The NBR used was JSRN230S (35% acrylonitrile content, Mooney viscosity of 42 ML (1 + 4) 100 °C), a Japanese JSR product (JSR Corporation, Tokyo, Japan), with the molecular formula illustrated in Figure 1a. The BIIR used was BB2255 (Mooney viscosity of 46 ML (1 + 4) 100 °C), an ExxonMobil Chemical Company product (Exxon Mobil Corporation, Ivan, Texas, USA), with the molecular formula illustrated in Figure 1b. The EVA copolymer used was 40 W, a US DuPont product (DuPont, Wilmington, Delaware, USA), with the molecular formula illustrated in Figure 1c. Carbon black (N330), sulfur (S), dicumyl peroxide (DCP), zinc oxide (ZnO), stearic acid, coumarone, dibutyl phthalate (DBP), N-cyclohexyl-2-benzothiazole sulfonamide (CZ), tetramethyl thiuram disulfide (TMTD), an antioxidant (4010NA), an anti-aging agent D (PBAN, C_16_H_13_N) and other reagents used were all commercially available products (Shanghai Deyin Chemical Company, Shanghai, China).

### 2.2. Basic Formulation

According to the design principle of rubber formula 3P (price + performance + process) +1C (circumstances), the basic formulation is designed by mathematical methods such as multi-factor optimization method (orthogonal experiment method) and variance analysis method. The basic formulation of NBR, BIIR and EVA masterbatch are shown in Table 1.

### 2.3. Sample Preparation

**Figure 2:**
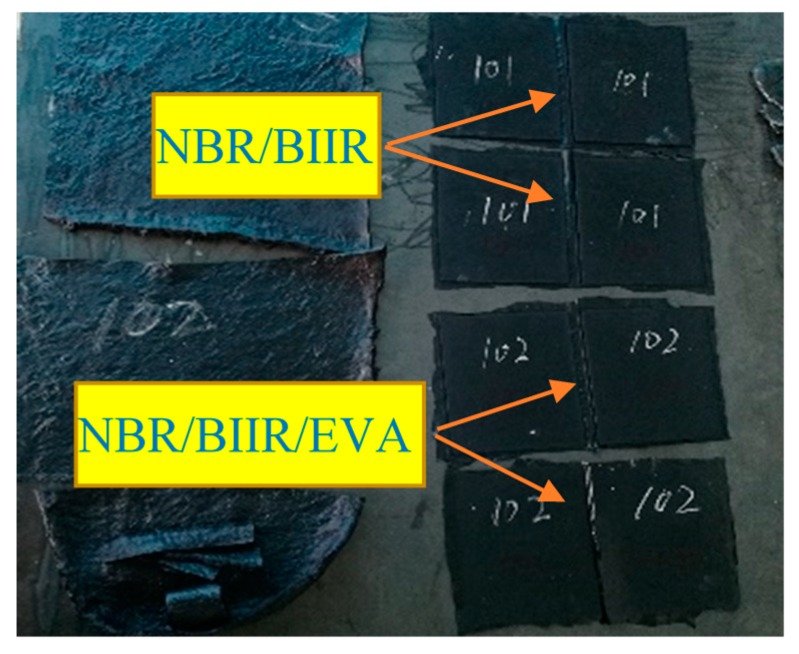
Test samples.

1. NBR, BIIR and EVA masterbatch: The as-received NBR, BIIR and EVA were kneaded on a two-roll mill at room temperature for 3 min and then blended with compounding and cross-linking additives (the basic formulas of the recipes are shown in Table 1), respectively. Each compound was then kneaded on the two-roll mill at room temperature for 5 min to form the NBR, BIIR and EVA masterbatch.
2. NBR/BIIR and NBR/BIIR/EVA blends: The NBR, BIIR and EVA masterbatch was mixed in the corresponding proportions (the specific proportions of the blends are shown in Section 4.1 and Section 4.2) in a certain order (i.e., for NBR/BIIR, first BIIR, then NBR; for NBR/BIIR/EVA, first BIIR, then NBR, and finally EVA) on the two-roll mill at room temperature for 5 min. After 16 h, a rheometer analyzer was used to determine the scorch time T_10_ and the vulcanization time T_90_ of the mixture. These mixtures were then hot pressed and vulcanized at 150 °C under a pressure of 15 MPa for T_90_ to obtain cross-linked NBR/BIIR and NBR/BIIR/EVA samples (Figure 2).

### 2.4. Performance Test

#### 2.4.1. Vulcanization Characteristics

The samples were tested on an M-3000A moving die rheometer analyzer (Gotech Testing Machines Inc, Taiwan, China). The test temperature was 150 °C, and the test time was 30 min based on the ISO 6502:2016.

#### 2.4.2. Mechanical Property Test

The samples were tested on a WDL-2500N universal tensile testing machine (Gotech Testing Machines Inc, Taiwan, China). The tensile test involved a standard sample, a tensile rate of 500 mm/min, and a test temperature of 23 °C based on the ISO 37:2017. A trouser tear test was carried out at a tensile rate of 100 mm/min according to ISO 34:2016.

#### 2.4.3. Dynamic Mechanical Analysis (DMA)

A TA Instruments Q800 dynamic mechanical analyzer (TA Instruments, Wilmington, Delaware, USA) was adopted to measure the damping performance of the sample in accordance with the ISO 4664-1:2011. The sample dimensions were 35 mm × 12 mm × 2 mm (length × width × thickness), the test temperature range was −60–60 °C, the frequency was 10 Hz, and the heating rate was 3 °C/min.

#### 2.4.4. Aging Characteristic Test

**Figure 3:**
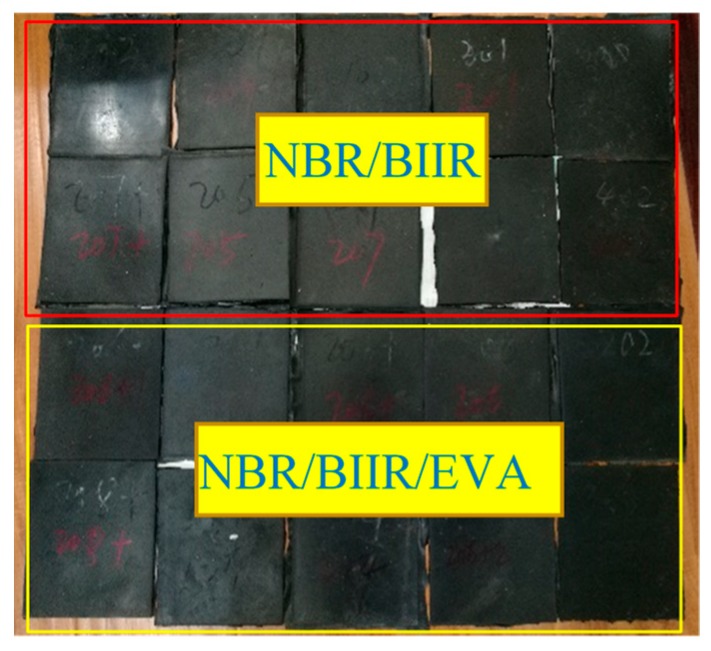
Samples after aging.

An R-PTH standard aging test box (Kerry Instruments Company, Shanghai, China) was used for accelerated aging of the pieces by heating in air according to ISO 188:2011. The aging condition was hot air at 100 °C × 72 h, as suggested by ISO 22762-3:2018. Sample performance was characterized based on the property variation percentage observed during aging (Figure 3).

#### 2.4.5. Shore Hardness Test

Sample preparation was made in accordance with ISO 23529: 2010. Hardness test was performed by using a Shore A durometer (LX-A; Jiangsu Mingzhu Testing Machinery, Yangzhou, Jiangsu, China) on the sample size of 15 mm × 15 mm × 6 mm according to ISO 48-4: 2018 at 25 ± 2 °C. Three measurements were involved at different positions on each side of a 6 mm thick specimen obtained by compression molding, and the median value was determined. The standard deviation for the Shore A hardness was 3%. The reading was taken 3 s after the pressure foot was in firm contact with the test piece.

## 3. Polymer Blend Theory

### 3.1. Phase Structure Mechanism of Polymer

The phase structure of a polymer is closely related to the compatibility of the basic materials. The better the compatibility between two basis materials is, the smaller the two-phases separation of the final material will be.

**Figure 4:**
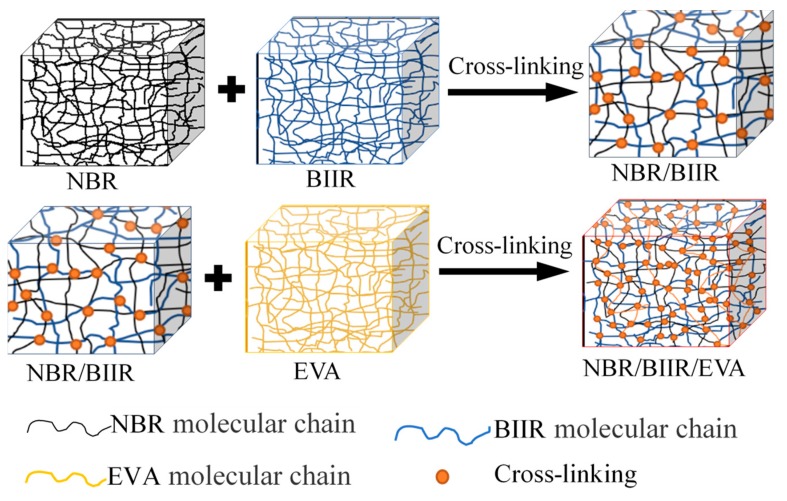
Basis blending and cross-linking mechanism.

NBR/BIIR was prepared by blending strongly polar NBR with weakly polar BIIR base rubber. EVA copolymer was added to NBR/BIIR to promote the compatibility between NBR and BIIR phases and to further improve the overall performance of NBR/BIIR blends. The basis blending and cross-linking mechanism are shown in Figure 4.

### 3.2. Polymer Wide Temperature Range Mechanism

**Figure 5:**
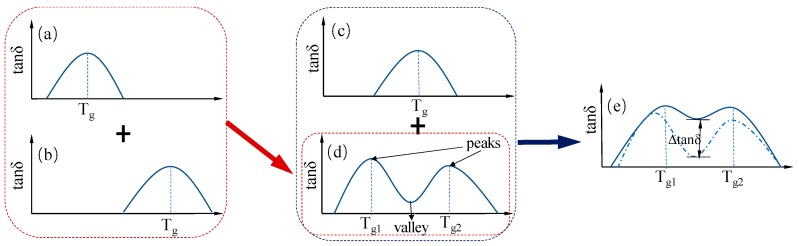
Variation of dynamic mechanical analysis (DMA) temperature spectrum of blends with miscibility of component polymers; (**a**–**c**) are the DMA temperature spectrum of the component high polymer, respectively; in (**d**,**e**), the miscibility increases sequentially.

Two slightly soluble rubbers, NBR and BIIR, were blended, one component being BIIR with a glass transition temperature of *T*_g1_ (see Figure 5a) and the other component being NBR with a glass transition temperature of *T*_g2_ (see Figure 5b). The tanδ-T curve of the blends shows two external damping internal friction peaks, effectively expanding the scope of the damping temperature range. However, due to the low compatibility between NBR and BIIR, the damping value is low in the valley between the two internal friction peaks (see Figure 5c). By adding EVA copolymer, a blend with a high-damping value over a wide temperature range can be prepared (see Figure 5d). The dynamic mechanical analysis (DMA) temperature profile of the blend system varies with the miscibility of the constituent polymers, as shown in Figure 5.

### 3.3. Polymer Damping Mechanism

NBR/BIIR and NBR/BIIR/EVA blends have different energy conversion modes (i.e., energy dissipation and energy storage) at different ambient temperatures during the deformation process. The entire polymer is in a glassy state when the temperature is below the lower limit of the glass transition range, and in a free state when above the upper limit of the glass transition range. In the two states, mechanical energy cannot be dissipated (i.e., energy storage). However, in the glass transition range, the chains of polymer molecules are in a critical state between “freezing” and “thawing,” also called a viscoelastic state. When subjected to an external force, the polymer follows the energy dissipation mechanism of the viscoelastic material.

## 4. Results and Discussion

### 4.1. NBR/BIIR Results and Discussion

#### 4.1.1. Fluidization Characteristics

**Figure 6:**
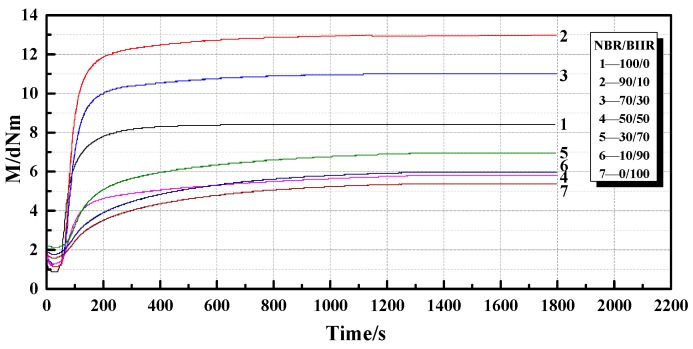
Curves of vulcanization characteristics for NBR/BIIR with various blend ratios.

The vulcanization curves and corresponding characteristics of NBR/BIIR with various blend ratios are shown in Figure 6 and Table 2.

As shown in Figure 6, when a low-sulfur and high-accelerator vulcanization system was adopted, NBR/BIIR blends with various blend ratios exhibited good vulcanization plateau. Table 2 shows that when the amount of BIIR was increased from 0 to 90 phr, the vulcanization time and scorch time first increased and then gradually stabilized. The main reason is that the vulcanization and scorch times of BIIR are longer than those of NBR. When the BIIR content exceeded 50 phr, BIIR played a dominant role in determining the properties of NBR/BIIR, and the vulcanization time of the blend was greatly extended. Additionally, with the increase in BIIR content, the maximum torque of the blend increased first and then decreased. When the NBR/BIIR ratio of the blend was 50/50, the minimum torque was 6.0 dNm. The reason is that NBR has a stronger intermolecular interaction than BIIR.

#### 4.1.2. Mechanical Properties

**Figure 7:**
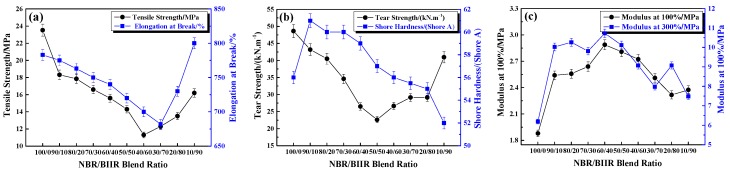
Mechanical properties of NBR/BIIR with various blend ratios: (**a**) Effect of tensile strength and elongation at break; (**b**) effect of tear strength and shore hardness and (**c**) effects of 100% and 300% module.

Mechanical properties, as indispensable performance indices of composite materials for high-damping-isolation bearings, must meet the requirements of ISO 22762-3:2010. The mechanical properties of NBR/BIIR with various blend ratios are shown in Figure 7.

With the increase in BIIR content, the tensile strength and the elongation at break of the NBR/BIIR varied as a “V-shaped” pattern (Figure 7a). When the content of BIIR was 60 phr, the tensile strength and the elongation at the break were the lowest, but still reached 11.2 MPa and more than 675%, respectively. First, BIIR is more difficult to vulcanize than NBR, and the difference between the two-phase vulcanization speeds leads to different crosslink densities. In addition, because of the poor thermodynamic compatibility of the two rubbers, weak links were formed at the interface between the two phases, which reduced the mechanical properties of the blends. These two reasons resulted in a decrease in tensile strength and elongation at break.

With increasing BIIR, the tear strength decreased first and then increased as a result of the joint action of two factors (Figure 7b). The addition of BIIR increased the number of polysulfide bonds in the blend system, which provides high tear strength. However, increasing the cross-linking density decreased tear strength. When the blend ratio was 50/50, the tear strength reached a minimum of 22.6 MPa.

The modulus and hardness are both representatives of the rigidity of the blends. The modulus is related to large tensile deformation, while the hardness is related to smaller compressive deformation. As shown in Figure 7b,c, with an increase in the BIIR content, the change trends of the modulus and the hardness were roughly the same, both increasing first and then decreasing.

#### 4.1.3. Damping Performance

**Figure 8:**
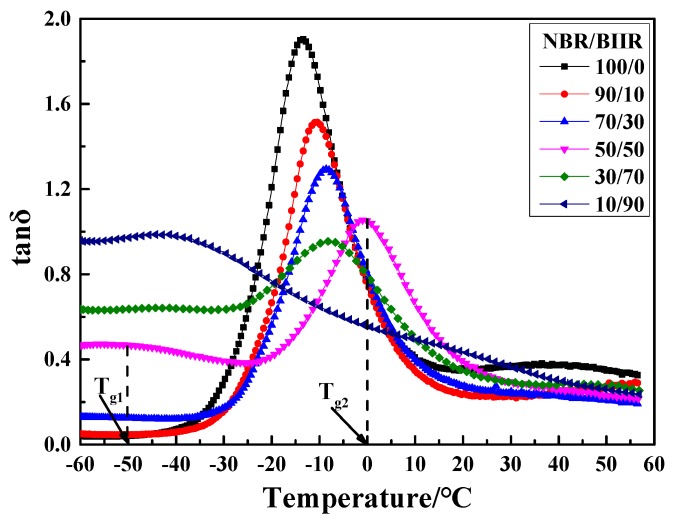
Damping properties versus temperature for NBR/BIIR with various blend ratios.

The dissipation factor tanδ-T curves and damping parameter of NBR/BIIR with various blend ratios are shown in Figure 8 and Table 3.

Figure 8 shows that, with increasing BIIR, two damping peaks appeared in the tanδ-T curve. The low-temperature peak and the high-temperature peak correspond to *T*_g1_ and *T*_g2_ of BIIR and NBR, respectively. Moreover, the effective damping temperature range gradually expanded, but the damping peak decreased. This phenomenon was due to the large difference in polarity between NBR and BIIR, resulting in a certain phase separation tendency. When NBR was blended with BIIR, the moving ability of the NBR molecular segments was limited by surrounding and entangling actions from the bulky BIIR molecular segments. However, the slightly miscible BIIR reduced the intermolecular interactions of NBR, thus increasing the movement capacity of the NBR molecular chains. The two effects caused the *T*_g_ of NBR to move toward high temperatures. However, when the BIIR content increased to a certain limit, *T*_g_ moved in the opposite direction. At this point, BIIR played a dominant role in the blend system.

Table 3 shows that, as the BIIR content increased, the effective temperature range expanded and moved toward low temperatures. When the NBR/BIIR ratio was 50/50, the effective temperature range was at least 87.0 °C, but the damping at the bottom in the tanδ-T curve was low. Therefore, it was necessary to further improve the damping in the effective temperature range.

**Figure 9:**
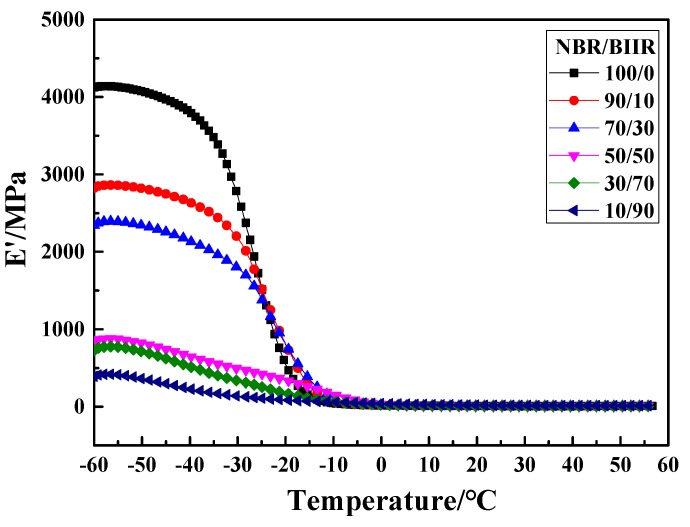
Storage modulus E′ versus temperature of NBR/BIIR with various blend ratios.

The storage modulus E′-T curves of NBR/BIIR are shown in Figure 9. The E′-T curves of both NBR/BIIR and pure NBR exhibit a transition peak. As the BIIR content increased, the storage modulus decreased significantly in the glassy region. When the BIIR content exceeded 50, the storage modulus tended to stabilize. This result shows that BIIR has a softening plasticizer effect on the NBR base rubber, making the NBR/BIIR blend softer and thus increasing the energy dissipation and improving the damping properties of the blend system.

#### 4.1.4. Aging Performance

The stability of the rubber material for rubber isolation bearings has a strong effect influence on the mechanical and damping performance of the bearings. Therefore, it was necessary to perform a further heat aging test based on the ISO 22762-3:2018.

1. Mechanical Properties

**Figure 10:**
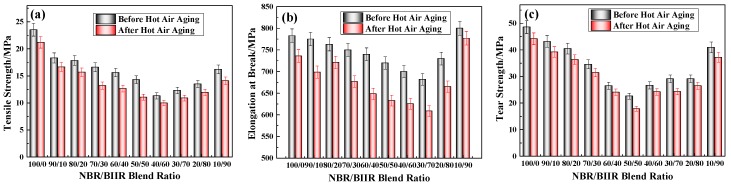
Comparison of mechanical properties before and after aging of NBR/BIIR with various blend ratios: (**a**) Tensile strength; (**b**) elongation at break, and (**c**) tear strength.

The mechanical properties and aging performance of NBR/BIIR with various blend ratios are shown in Figure 10 and Table 4.

Figure 10 shows that the various mechanical properties of the NBR/BIIR blend decreased after aging and that the change trends of the mechanical properties with the change of BIIR content were roughly the same as those before. When the blend ratio was 50/50, the tensile strength and tear strength decreased by 22.7% and 16.3%, respectively, and the corresponding elongation at break decreased by 12.1% (Table 4). In general, the overall stability of the blends was more ideal.

2. Damping Performance

**Figure 11:**
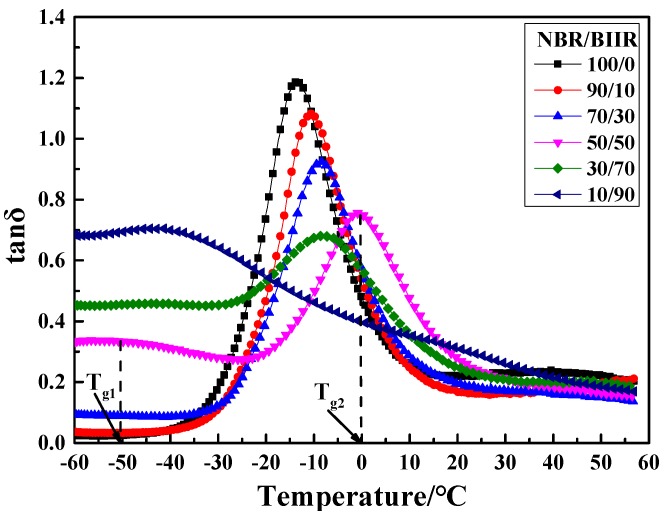
Damping properties versus temperature for NBR/BIIR with various blend ratios.

A DMA test was further performed after hot air thermal aging of the NBR/BIIR materials. The damping performance of the NBR/BIIR blends are shown in Figure 11.

The tanδ peak of NBR/BIIR with various blend ratios after aging was slightly lower than that before aging (Figure 8). Moreover, the overall change trend remained essentially consistent with that before aging. The rubber damping performance shows good stability for various ratios. When the blend ratio of NBR/BIIR was 50/50, the blend could meet the requirement of tanδ ≥ 0.3 and showed a wide temperature range of no less than 75.6 °C.

### 4.2. NBR/BIIR/EVA Results and Discussion

Based on the tests performed, we can conclude that when the NBR/BIIR blend ratio is 50/50, the blend achieves the best performance (mechanical and damping properties). However, the effective damping temperature region is concentrated in the low-temperature zone, and the damping in the valley of the tanδ-T curve is low.

To obtain a more ideal blend with a wider temperature range and higher damping, EVA copolymer was added to the blend with an NBR/BIIR blend ratio of 50/50. The effect of the EVA copolymer on the properties of NBR/BIIR is discussed in detail.

#### 4.2.1. Fluidization Characteristics

**Figure 12:**
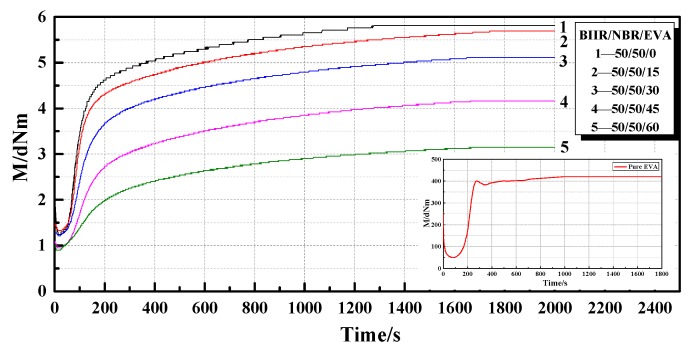
Curves of vulcanization characteristics for NBR/BIIR/EVA with various blend ratios.

The vulcanization curves of NBR/BIIR (50/50) after adding the EVA copolymer are shown in Figure 12 and Table 5.

As shown in Figure 12, NBR/BIIR/EVA exhibited good vulcanization plateau at various blend ratios. The addition of EVA decreased the maximum torque of the blends (Figure 6). Table 5 shows that with increasing EVA content, *T*_90_ increased. When the EVA content was 0 and 60 phr, the corresponding *T*_90_ values were 13.2 and 17.6 min, respectively.

This phenomenon is due to the different mutual cross-linking speeds caused by the different sensitivities of the EVA copolymer and the NBR/BIIR to the vulcanizing agent, thus resulting in different vulcanization times. As the EVA content increased, the rate of cross-linking within NBR/BIIR blends was further impeded, and the vulcanization rate of the blend was weakened.

#### 4.2.2. Mechanical Properties

**Figure 13:**
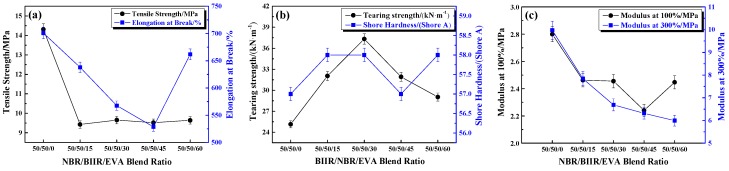
Mechanical properties of NBR/BIIR/EVA with various blend ratios: (**a**) Effect of tensile strength and elongation at break; (**b**) effect of tear strength and shore hardness, and (**c**) effects of 100% and 300% module.

The effects of various blend ratios on the mechanical properties of NBR/BIIR/EVA are shown in Figure 13.

Figure 13a shows that the tensile strength of the blend decreased when a small amount of EVA was added. As the EVA content was increased, the tensile strength remained nearly stable, and the minimum was 9.3 MPa. The elongation at break varied according to a “V-shaped” pattern. The minimum was 525% when the NBR/BIIR/EVA blend ratio was 50/50/45. The main reason is that the tensile strength and elongation at break of the EVA copolymer are lower than those of the rubber material, and the difference in the vulcanization rates results in an inconsistent crosslink density.

With increasing EVA, the tear strength increased first and then decreased as shown in Figure 13b. The addition of EVA increased the number of polysulfide bonds, the crosslink density and intermolecular force, which increased the tear strength of the blend. When the NBR/BIIR/EVA ratio was 50/50/30, the maximum tear strength reached 37.5 MPa.

As the EVA content increased, the modulus decreased and the hardness tended to stabilize, as shown in Figure 13c. Although the addition of EVA copolymer leads to a loss of mechanical properties, the resulting material could fulfill the requirements according to ISO 22762-3:2010.

#### 4.2.3. Damping Performance

**Figure 14:**
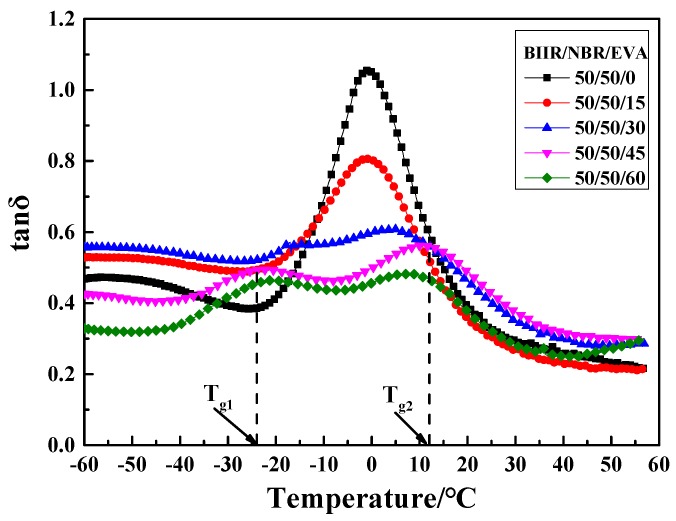
Damping properties versus temperature for NBR/BIIR/EVA with various blend ratios.

Further tests on the effect of the EVA copolymer on the dynamic mechanical properties of the NBR/BIIR blends were performed. The tanδ-T curve and damping parameters of the NBR/BIIR/EVA blend with various blend ratios are shown in Figure 14 and Table 6, respectively.

When the blend ratio of NBR/BIIR/EVA was 50/50/15, two peaks appeared in the tanδ-T curve. With increasing EVA, the damping peaks continuously decreased. The peak of *T*_g2_ moved toward a higher temperature, which effectively increased the damping in the high-temperature zone. The damping in the valley increased first and then decreased. When the EVA content was 30 phr, the damping in the valley reached up to 0.5, much higher than the former value of 0.4 observed for the NBR/BIIR (Figure 8).

Table 6 shows that when the ratio of NBR/BIIR/EVA was 50/50/45, the effective damping temperature range reached at least 89.5 °C (from −60 to 29.5 °C). The addition of EVA expanded the effective damping temperature range of the blend and effectively increased the damping at the valley of the tanδ-T curve (Table 3). One reason is that the EVA copolymer exhibits good compatibility with the basis rubbers. EVA enhances the solubility of NBR and BIIR, and strongly affects the interaction of the basic material. Thus, the phase structure of the blend changes from an “island” phase structure to a continuous phase, with good compatibility.

**Figure 15:**
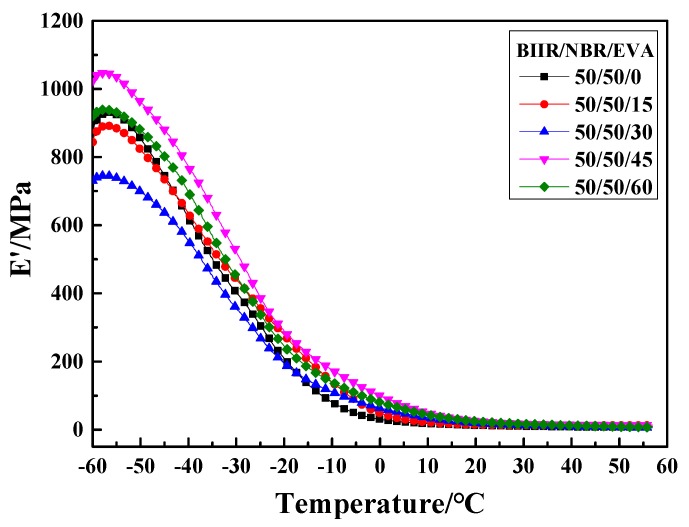
Storage modulus E′ versus temperature of NBR/BIIR/EVA with various blend ratios.

The storage modulus E′-T curves of NBR/BIIR/EVA are shown in Figure 15. The E′-T curves of NBR/BIIR/EVA all show transition peaks. As the ratio of EVA reached a certain value, the E′ peak changes remained relatively stable in the glassy region, indicating that the plasticizer of EVA was no longer distinct.

#### 4.2.4. Aging Performance

Due to the mutual influence of the basis rubber, and the influence of the vulcanization system on aging, it was necessary to further study the anti-aging stability of the NBR/BIIR/EVA blends.

1. Mechanical properties

**Figure 16:**
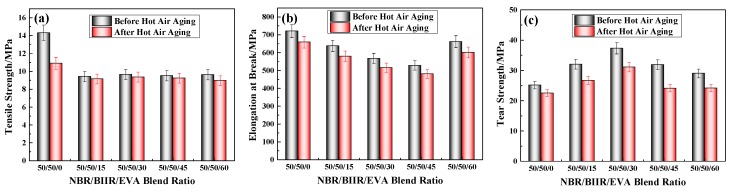
Comparison of mechanical properties before and after aging of NBR/BIIR/EVA with various blend ratios: (**a**) Tensile strength; (**b**) elongation at break, and (**c**) tear strength.

The mechanical properties of NBR/BIIR/EVA before and after hot air thermal aging tests are compared in Figure 16 and Table 7.

As shown in Figure 16, the mechanical properties of the NBR/BIIR/EVA blends deteriorated after the hot air thermal aging test, and the overall changing trends after aging remained essentially similar to those before. Table 7 shows that, with the addition of EVA, the aging resistance of tensile strength was significantly improved, but the effect on tear strength and elongation at the break was not distinct (Table 4). The maximum deterioration in the tensile strength, elongation at break and tear strength were 6.9%, 10.2%, and 21.3%, respectively. Nevertheless, all of the results demonstrate that the blends exhibited strong anti-aging properties.

2. Damping Performance

**Figure 17:**
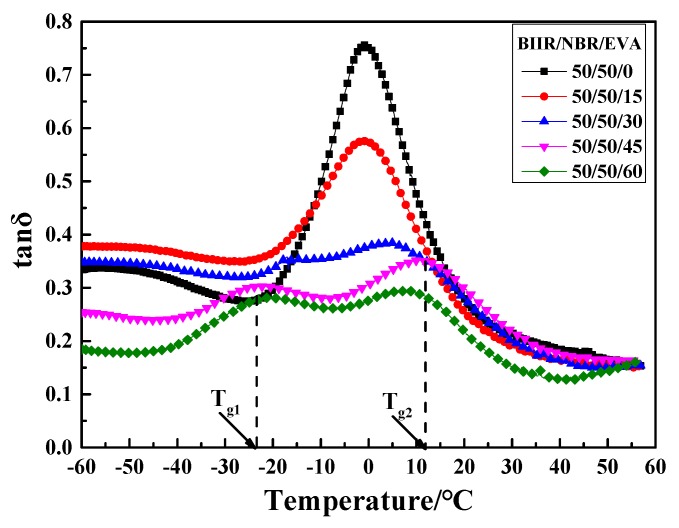
Damping properties versus temperature of NBR/BIIR/EVA with various blend ratios.

Additionally, a DMA test was performed, and the damping performance is shown in Figure 17.

Due to the degradation of the material and the environmental conditions, the damping performance of the blends was reduced. The overall change was similar to that before aging, but the temperature range was not significantly reduced. As the EVA content was increased, the damping aging resistance of the blend decreased.

## 5. Conclusions

Based on tests of the blends NBR/BIIR and NBR/BIIR/EVA, the following conclusions were drawn:

- NBR/BIIR and NBR/BIIR/EVA possess good vulcanization and mechanical properties. The addition of EVA reduces the mechanical properties of NBR/BIIR. Nevertheless, the blends can still fulfill the standard requirements.
- The blending of NBR and BIIR is efficient in expanding the damping temperature range. When the NBR/BIIR ratio is 50/50, the effective temperature range is at least 87.0 °C. However, the damping in the valley is low.
- The addition of EVA to the NBR/BIIR effectively increases the damping in the valley of the tanδ-T curve (tanδ ≥ 0.3) and moves the effective temperature range toward high temperatures. Moreover, the NBR/BIIR/EVA with a 50/50/30 blend ratio is a comparatively ideal material.
- Hot air thermal aging tests of NBR/BIIR and NBR/BIIR/EVA show that the anti-aging stabilities of the two materials are not significantly altered.

## Figures and Tables

**Table 1:** Basic formulation of nitrile rubber (NBR), brominated butyl rubber (BIIR) and brominated butyl rubber (EVA) (100 phr).

Name	ZnO	Stearic Acid	S	DCP	2402 Resin	TMTD	CZ	DBP	N330	PBAN	4010NA
NBR	5–10	2–6	1–4	–	–	0.2–0.6	1–3	8–10	20–50	1.5–3	1–3
BIIR	5–10	2–6	–	–	5–10	0.2–0.4	1–1.5	8–10	20–50	1.5–3	1–3
EVA	5–10	2–6	–	1–2	–	0.2–0.4	1–1.5	8–10	20–50	1.5–3	1–3

**Table 2:** Vulcanization characteristics of NBR/BIIR with various blend ratios (150 °C).

Test Numbers	Rubber Name NBR/BIIR	MH dNm	ML dNm	*T*_10_ min	*T*_90_ min	tanPA (min)
1	100/0	8.5	0.9	0.6	3.2	0.08
2	90/10	13.0	1.2	1.1	3.1	0.03
3	70/30	11.1	1.4	1.1	3.4	0.04
4	50/50	6.0	1.3	1.0	13.2	0.07
5	30/70	7.1	2.0	1.1	12.5	0.08
6	10/90	6.1	1.8	1.2	13.2	0.05
7	0/100	5.4	1.6	1.2	13.5	0.05

Note MH: Maximum torque rating; ML: Minimum torque rating; *T*_10_: Scorch time; *T*_90_: Optimal vulcanization time; tanPA: Dynamic dissipation angle.

**Table 3:** Damping property parameters of NBR/BIIR with various blend ratios.

NBR/BIIR	Value	*T*_g_ ( = *T*_g2_)/ °C	*T*_1_/ °C	*T*_2_/ °C	(*T*_2_ − *T*_1_)/ °C
100/0	—	−14.3	−29.8	18.1	47.9
90/10	—	−8.3	−25.9	12.0	37.9
70/30	—	−5.1	−24.9	15.9	40.8
50/50	0.4	0.4	≤−60.0	27.0	≥87.0
30/70	0.6	−5.8	≤−60.0	25.6	≥85.6
10/90	1.0	−38.9	≤−60.0	38.5	≥98.5

Note: Values are those observed in the valley of the tan δ-*T* curve; *T*_1_ and *T*_2_ are the lowest and highest temperatures that satisfy tanδ ≥ 0.3, respectively; and *T*_2_−*T*_1_ is the effective temperature range.

**Table 4:** Mechanical property parameters before and after aging of NBR/BIIR with various blend ratios.

NBR/BIIR	Tensile Strength/MPa	Elongation at Break/%	Tear Strength/kN/m
	Before and after Aging/%	Before and after Aging/%	Before and after Aging/%
100/0	9.9 ↓	6.0 ↓	9.1 ↓
90/10	9.1 ↓	9.9 ↓	9.1 ↓
80/20	12.2 ↓	5.5 ↓	10.2 ↓
70/30	20.4 ↓	9.8 ↓	9.1 ↓
60/40	18.8 ↓	12.4 ↓	9.2 ↓
50/50	22.7 ↓	12.1 ↓	16.3 ↓
40/60	11.8 ↓	10.7 ↓	9.6 ↓
30/70	11.4 ↓	10.7 ↓	15.4 ↓
20/80	11.5 ↓	8.9 ↓	10.7 ↓
10/90	13.6 ↓	2.9 ↓	9.0 ↓

Note: Property variation percentage during aging = (after aging – before aging)/before aging 100%; “↓” indicates decrease.

**Table 5:** Vulcanization characteristic parameters of NBR/BIIR/EVA with various blend ratios (150 °C).

Test Number	Rubber Name NBR/BIIR/EVA	MH dNm	ML dNm	*T*_10_ min	*T*_90_ min	tanPA (min)
1	50/50/0	6.0	1.3	1.0	13.2	0.07
2	50/50/15	5.7	1.3	1.1	14.3	0.07
3	50/50/30	5.3	1.2	1.1	15.2	0.07
4	50/50/45	4.3	1.0	1.1	17.0	0.07
5	50/50/60	3.3	0.9	1.0	17.6	0.10
6	0/0/100	421	49.5	1.3	2.3	0.03

Note MH: Maximum torque rating; ML: Minimum torque rating; *T*_10_: Scorch time; *T*_90_: Optimal vulcanization time; tanPA: Dynamic dissipation angle.

**Table 6:** Damping property parameters of NBR/BIIR/EVA with various blend ratios.

NBR/BIIR/EVA	Value	*T*_g_( = *T*_g2_)/ °C	*T*_1_/ °C	*T*_2_/ °C	(*T*_2_ − *T*_1_)/ °C
50/50/0	0.4	0.4	≤−60.0	27.0	≥87.0
50/50/15	0.5	3.2	≤−60.0	25.1	≥85.1
50/50/30	0.5	8.9	≤−60.0	27.9	≥87.9
50/50/45	0.4	12.5	≤−60.0	29.5	≥89.5
50/50/60	0.3	11.5	−48.6	21.7	69.4

Note: Values are those observed in the valley of the tan δ-T curve; *T*_1_ and *T*_2_ are the lowest and highest temperatures that satisfy tanδ ≥ 0.3, respectively; and *T*_2_ − *T*_1_ is the effective temperature range.

**Table 7:** Mechanical property parameters before and after aging of NBR/BIIR/EVA with various blend ratios.

NBR/BIIR/EVA	Tensile Strength/MPa	Elongation at Break/%	Tear Strength/ kN/m
	Before and after Aging/%	Before and after Aging/%	Before and after Aging/%
50/50/0	22.7↓	12.1↓	16.3↓
50/50/15	2.9↓	9.1↓	16.2↓
50/50/30	2.9↓	10.2↓	15.6↓
50/50/45	3.0↓	10.3↓	21.3↓
50/50/60	6.9↓	9.0↓	15.5↓

Note: Property variation percentage during aging = (before aging − after aging)/ before aging 100%, “↓” indicates decrease.
